# Plasma YKL-40 and NGAL are useful in distinguishing ACO from asthma and COPD

**DOI:** 10.1186/s12931-018-0755-6

**Published:** 2018-03-27

**Authors:** Jing Wang, Huajie Lv, Zhuang Luo, Shan Mou, Jing Liu, Chang Liu, Shiying Deng, Youfan Jiang, Jiachen Lin, Chengzhou Wu, Xianhong Liu, Jinzhi He, Depeng Jiang

**Affiliations:** 1 Department of Respiratory Medicine, the Second Clinical Hospital of Chongqing Medical University, Chongqing, 400010 China; 2 Department of Respiratory Medicine, the First Clinical Hospital of Kunming Medical University, Kunming, 650032 Yunnan Province China; 3 Department of Laboratory Medicine, the Second Clinical Hospital of Chongqing Medical University, Chongqing, 400010 China; 4 Department of Respiratory Medicine, Chengdu Second People’s Hospital, Chengdu, 610011 Sichuan Province China; 5 Department of Respiratory Medicine, People’s Hospital of Wuxi Country, Chongqing, 405800 China; 6 Department of Respiratory Medicine, People’s Hospital of Shizhu Country, Chongqing, 409100 China; 7 Department of Respiratory Medicine, People’s Hospital of Fengjie Country, Chongqing, 404600 China

**Keywords:** Asthma COPD overlap (ACO), Chronic obstructive pulmonary disease, Asthma, YKL-40, NGAL

## Abstract

**Background:**

Asthma-chronic obstructive pulmonary disorder (COPD) overlap (ACO) is characterized by the coexistence of features of both asthma and COPD and is associated with rapid progress and a poor prognosis. Thus, the early recognition of ACO is crucial.

**Objectives:**

We sought to explore the plasma levels of biomarkers associated with asthma (periostin, TSLP and YKL-40), COPD (NGAL) and their possible correlation with lung function, the bronchodilator response and radiographic imaging in patients with asthma, COPD and with features of ACO.

**Methods:**

We enrolled 423 subjects from 6 clinical centers. All participants underwent blood collection, lung function measurements, bronchodilator response tests and high-resolution CT. Correlations of the plasma biomarkers with lung function, the bronchodilator response and percentemphysema were calculated by Spearman’s rank correlation and multivariate stepwise regressionanalysis.

**Results:**

1) Patients with features of ACO had lower plasma YKL-40 than COPD patients and a moderate elevated plasma level of NGAL compared with asthma patients. 2) Patients with features of ACO had an intermediate degree of airflow obstruction, the bronchodilator response and emphysema between patients with COPD and asthma. 3) Plasma YKL-40 was negatively correlated with lung function and with the bronchodilator response, and plasma NGAL was positively correlated with the extent of emphysema.

**Conclusions:**

Plasma YKL-40 is a promising candidate for distinguishing between patients with features of ACO and COPD patients, while plasma NGAL may be a valuable biomarker for differentiating between patients with features of ACO and asthma patients.

**Clinical trial registration:**

ChiCTR-OOC-16009221.

## Background

Asthma and chronic obstructive pulmonary disease (COPD) are common pulmonary diseases with significant impacts on public health throughout the world [1–3]. There is a long-standing debate about whether asthma and COPD are manifestations of the same disease or completely distinct disease entities generated by different mechanisms [4]. It is generally accepted that COPD and asthma differ from each other in their patterns of inflammation, pathophysiology mechanisms and extent of the reversibility of airflow limitation [1, 5]. However, some patients may present with features of both diseases, making it difficult to differentiate between asthma and COPD and confusing the clinical treatment. To discriminate this condition, the term asthma–COPD overlap (ACO) has been recommended to describe patients who have features of COPD and asthma [6, 7].

There is increased awareness of the importance of recognizing ACO because it is associated with more frequent and severe exacerbations, a rapid decline in pulmonary function, poor quality of life and higher mortality than asthma or COPD alone [8, 9]. In addition, its better response to inhaled corticosteroids has led to the recommendation of the early introduction of corticosteroids in these patients [8, 10]. It is therefore very important to make an early recognition of ACO.

To date, the universally accepted definition of ACO remains elusive. In fact, the definition of ACO is evolving, and different definitions are being applied in various studies [11–13]. ACO is characterized with persistent airflow limitation that has several features associated with asthma and several features associated with COPD. As asthma and COPD are heterogeneous diseases with a range of underlying mechanisms respectively, ACO is no longer been considered as a single discrete disease entity, and the lack of a unified definition and diagnostic criteria makes the identification of ACO challenging and understanding of the pathophysiology difficult.

Previous studies have demonstrated that asthma, COPD and ACO differ in their biomarker profiles [14–16], thus we hypothesized that biomarker measurements are an important aspect in the recognition of ACO, which coupled with clinical features, spirometry and imaging would hold great promise for identifying ACO patients. However, there are limited published studies on biomarkers for ACO.

It is generally accepted that ACO has two clinical phenotypes: asthma with long-term smoking developed non-fully reversible airflow obstruction in elderly adults and COPD accompanied by reversible or partially reversible airflow obstruction with a history of asthma or “asthmatic” symptoms [17, 18].Based on the overlapping features of asthma and COPD, we hypothesized that biomarkers associated with airway inflammation in asthma or COPD would be helpful to clinically identify ACO. In the present study, we investigated the plasma levels of four potential biomarkers in asthma and COPD patients and patients with features of ACO to investigate the inflammatory features of ACO. Among them, periostin [19] and TSLP [20] are well-characterized T-helper 2 type markers with specific roles in various stages of asthma pathogenesis. Neutrophil gelatinase-associated lipocalin (NGAL) is a well-characterized neutrophil-derived inflammatory molecule associated with smoke-related airway inflammation and pulmonary parenchyma injury [21]. YKL-40, originally shown to be an upstream cytokine that drives dendritic cells to enhance Th2 polarization in asthmatic patients [20, 22], is now found to be more related with remodeling and more elevated in COPD patients [23, 24], suggesting its wide-ranging biology more than Th2 inflammation.

Previous studies have revealed several key features of ACO, including the intermediate degree of emphysema and airflow obstruction between asthma and COPD and mixed, moderate airway neutrophil and eosinophil infiltration [11, 18]. We hypothesized that patients with features of ACO present a characteristic biomarker profile that could reflect its underlying pathological essence, and the measurements of these objective biomarkers would be used to improve the determination and recognition of ACO. To test our hypothesis, we further explored the potential correlation between the above selected biomarkers and pathological changes in airway remodeling and lung parenchyma destruction.

In our study, ACO had significantly lower plasma levels of YKL-40 than COPD and an intermediate degree of plasma NGAL, airflow obstruction and emphysema between COPD and asthma. Additionally, univariate and multivariate stepwise analyses displayed that both YKL-40 and NGAL were correlated with airflow limitation and an impaired bronchodilator response, and NGAL was independently correlated with the degree of emphysema. Our results strongly suggest that YKL-40 is a promising candidate for identifying ACO from COPD, while plasma NGAL is helpful in distinguishing ACO from asthma.

## Methods

### Subjects

This study was part of a multicenter case-control study hosted by the Department of Respiratory Medicine, the 2nd clinical hospital of Chongqing Medical University, China. Additionally, 6 respiratory medicine departments at other public hospitals participated in this study. We recruited 423 participants: 147 with COPD, 124 with asthma, 102 with AOCS and 50 healthy nonsmokers. All participants were Chinese who presented to the inpatient department of these hospitals from January 2016 to May 2017.

At the time of enrollment, all subjects completed standardized questionnaires regarding sex, age, BMI, smoking habits, medication use, and exacerbation history. Simultaneously, all subjects, except for the healthy nonsmokers, completed the St. George’s Respiratory Questionnaire (SGRQ). All subjects underwent spirometry, high-resolution computed tomography (HRCT) and blood collection on the same day. The diagnosis of asthma and COPD was based on the GINA [25] and GOLD [6] guidelines respectively. The diagnosis of ACO was made when patients met either one of two criteria: (1) of the patients with asthma, those aged ≥40 years who had been cigarette smokers (greater than 10 pack-years) or had significant biomass exposure (wood or coal for cooking and heating; exposure ≥100 h/year) demonstrated persistent airflow limitation defined as a post-bronchodilator FEV1/FVC < 0.7; (2) of the patients with COPD, those had a history of physician-diagnosed asthma before the age of 40 or with a atopic status demonstrated reversible airway obstruction defined as a improvement in FEV1 > 200 ml and > 12% following bronchodilator administration.

Control subjects were recruited from healthy nonsmokers and no history of biomass exposure with normal spirometry (FEV1 ≥ 80% predicted and FEV1/FVC ≥0.7). The subjects were excluded from the study if they: (1) had a respiratory tract infection in the 2 weeks preceding the study; (2) had coexisting pulmonary diseases, such as bronchiectasis, cystic fibrosis, interstitial lung disease, or lung cancer; and 3) had any cognitive disorder or impairment in renal or liver function .

### Lung function measurements and bronchodilator response testing

Spirometry was performed on a computerized spirometer (MasterScreen, Leibnizstrasse, Hoechberg, Germany) in accordance with the American Thoracic Society and European Respiratory Society (ATS/ERS) recommendations. The examination included pre- and post-bronchodilator spirometry. The parameters collected were FVC, FEV1, percent predicted values of these parameters (%FVC, %FEV1) and the FEV1/FVC ratio. The bronchodilator response was quantified in two ways: as an absolute number (absolute, Change in FEV1(ml) and as a percent of the pre-bronchodilator value (relative, %Change in FEV1 (%)).

### Plasma sample collection and measurements of YKL-40, periostin, TSLP and NGAL

Peripheral whole venous blood was separately collected into EDTA tubes to measure various biomarkers. Plasma was prepared by centrifugation for 10–15 min at 4500 rpm and was stored at − 80 °C until analysis. YKL-40, periostin, TSLP and NGAL levels were measured using commercially available enzyme-linked immunosorbent assay (ELISA) kits (CUSABIO, Wuhan, China) according to the manufacturer’s instructions.

Neutrophil and eosinophil counts of peripheral blood were detected using a routine blood test and serum level of creatinine was measured using enzymatic methods.

### Evaluation of the degree of emphysema with HRCT scanning

Chest HRCT was performed at maximal inspiration, with the participants in the supine position using a 64-slice spiral CT scanner (Aquilion One, Toshiba Medical Systems Corporation, Otowara, Japan). The HRCT images were reconstructed with a window setting appropriate for the lungs (window level from − 700 to − 900 HU; width, from 800 to 1000 HU), with a 1-mm slice thickness using a high spatial frequency algorithm. All CT images were analyzed using 3D Slicer software (http://www.slicer.org). The degree of emphysema was defined as the percentage of lung voxels with attenuation values less than or equal to − 950 Hounsfield units.

### Statistics

All statistical analyses were performed with the SPSS 22.0 software program (IBM Corporation, Armonk, NY, USA) and *p* values < 0.05 were considered significant. Because the data of plasma biomarkers in our study were not normally distributed according to the Kolmogorov-Smirnov test, they were presented as the medians and interquartile ranges and comparisons between groups were evaluated using Kruskal–Wallis test followed by Dunn-Bonferroni post hoc comparison. The remaining data were presented as the means ± standard deviation (SD) and were analyzed with repeated measures ANOVA and unpaired t-test. Receiver operating characteristic (ROC) curves were obtained to elucidate the predictive capability of plasma biomarkers in distinguishing ACO from COPD and asthma. The correlations of lung function, the bronchodilator response and percent emphysema with the plasma biomarkers were calculated by Spearman’s rank correlation, linear regression was used to adjust plasma biomarkers level for age, BMI and smoke, and multivariate stepwise regression analysis was constructed to examine the independent variables on the plasma biomarker levels.

## Results

### Subject characteristics

The demographics and clinical characteristics of the subjects are shown in Table 1. Patients with features of ACO were younger than COPD patients but older than asthma patients. The pulmonary function parameters, including FVC% predicted, FEV1% predicted, the FEV1/FVC ratio and reversibility in the bronchodilator test, were lower in patients with features of ACO than in asthma patients but greater than in COPD patients. Additionally, the extent of emphysema in patients with features of ACO was between that in patients with asthma and COPD (Table 1). The patients with features of ACO had a higher BMI than asthma or COPD patients. Patients with features of ACO and COPD patients were more likely to be smokers and had more pack-years of smoking than control and asthma subjects. Peripheral blood neutrophil counts were highest in COPD groups and lowest in control and asthma groups, while eosinophil counts were highest in asthma group and lowest in control and COPD groups. Serum level of creatinine was similar among all the groups. There was no significant difference in the acute exacerbations per year among patients in different groups. In univariate analysis, subjects with COPD and features of ACO had a worse quality of life and more dyspnea than those with asthma as evidenced by higher SGRQ scores.

**Table 1:** Characteristics of the subjects

	Control(50)	Asthma(124)	ACOS(102)	COPD(147)
Age, years	49.60 ± 18.14	48.49 ± 12.25	62.54 ± 9.07	67.56 ± 9.18
BMI,kg.m^−2^	23.50 ± 1.11	24.43 ± 1.67	25.55 ± 1.21	22.43 ± 1.56
Smoker(former/current/never), n	0/0/50	22/49/53	14/78/10	34/102/11
Pack-years	/	13.62 ± 13.98	46.51 ± 15.78	39.31 ± 14.34
Sex(female/male), n	22/28	69/55	62/40	48/99
Years of biomass exposure≥10 (≥100 h/year)	0	23	45	72
Postbronchodialator				
FEV1, L	2.63 ± 0.23	2.10 ± 0.16	1.97 ± 0.17	1.67 ± 0.23
FVC, L	3.22 ± 0.25	2.77 ± 0.22	3.01 ± 0.26	2.66 ± 0.33
FEV1% pre	95.40 ± 7.65	73.67 ± 5.53	70.85 ± 5.62	58.95 ± 9.05
FVC% pre	98.02 ± 7.10	81.42 ± 6.91	80.49 ± 3.25	74.04 ± 6.72
FEV1/FVC	81.69 ± 4.48	75.91 ± 4.01	65.24 ± 2.86	62.65 ± 3.34
Response to bronchodilator				
% Change in FEV1,%	2.83 ± 1.04	16.91 ± 1.83	15.16 ± 1.04	6.26 ± 1.24
Change in FEV1,ml	72.42 ± 27.78	303.68 ± 37.70	259.64 ± 30.68	99.10 ± 26.31
SQRG score	/	39.51 ± 4.32	44.65 ± 3.66	52.27 ± 3.86
%LAA-950, %	3.24 ± 1.31	6.85 ± 2.26	11.49 ± 2.99	21.52 ± 5.21
Blood eosinophils, 10^8^/L	2.45 ± 1.11	3.45 ± 0.76	3.05 ± 0.87	2.75 ± 1.30
Blood neutrophil, 10^9^/L	3.59 ± 1.57	3.94 ± 1.43	4.43 ± 1.31	5.63 ± 2.08
Serum creatinine (umol/L)	73.40 ± 20.8	76.79 ± 15.94	78.08 ± 19.02	75.22 ± 15.38

Notes: Data are presented as the mean ± SEM or n (%)

*Abbreviations*: *ACO* asthma–COPD overlap, *COPD* chronic obstructive pulmonary disease, *BMI* body mass index, *FVC* forced vital capacity, *FEV1* forced expiratory volume in 1 s, *% pred* % predicted, *SGRQ* St George’s Respiratory Questionnaire, *%LAA-950* The percentage of the low attenuation area below − 950 HU

### Plasma levels of YKL-40, NGAL, TSLP and periostin in COPD, asthma and ACO

**Fig. 1:**
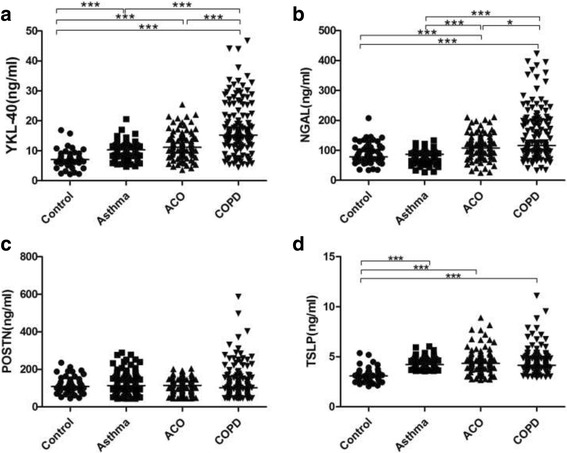
Plasma levels of **a**) YKL-40, **b**) NGAL, **c**) POSTN and **d**) TSLP in healthy control subjects and patients with asthma, ACO and COPD. Note:*: *p* < 0.05; **: *p* < 0.01; ***: *p* < 0.001. Abbreviations: ACO: asthma–COPD overlap; COPD: chronic obstructive pulmonary disease; YKL-40:chitinase-3-like protein 1; NGAL: neutrophil gelatinase-associated lipocalin, POSTN: Periostin;TSLP: Thymic stromal lymphopoietin

The results are presented as the medians (interquartile ranges, ng/ml). Compared with the plasma YKL-40 levels in healthy control subjects (7.08 [6.17–7.99] ng/ml), levels were significantly elevated in patients with asthma (10.33 [8.79–12.17] ng/ml, *p* < 0.0001) and those with features of ACO (11.13 [9.06–14.07] ng/ml, *p* < 0.0001) and were highest in patients with COPD (15.23 [12.31–20.72) (Fig. 1a). There was no significant difference in the levels between the asthma and ACO groups (*p* = 0.07).

We further performed regression analyses to ensure that the relationship between disease group and plasma biomarkers could be assessed after adjustment for potentially confounding variables, we found that after adjustment for all the subjects characterstics parameters (age, BMI, smoke, sex, blood eosinophil and neutrophil counts, serum creatinine, FEV1% pre, FVC % pre, FEV1/FVC, %Change in FEV1, degree of emphysema and SQRG scores) the statistically significant difference above remained markedly (*p* < 0.001).

Regarding the plasma level of NGAL, all three patient groups were significantly different from each other (Fig. 1b*, p* < 0.012). The difference between the control (78.59 [62.55–110.39] ng/ml) and asthma (87.33 [71.92–95.43] ng/ml) groups was not statistically significant(*p* = 0.90), and both groups showed significantly lower levels than those in the COPD (114.49 [96.05–202.27] ng/ml, *p* < 0.0001) and ACO (112.87 [77.30–149.56] ng/ml, *p* < 0.0002) groups.

Also, following regression analyses with adjustment for all the subjects characterstics parameters, patients with COPD and features of ACO both still had significantly higher NGAL levels than patients with asthma (*p* < 0.024), and a significant difference also remained between patients with features of ACO and COPD (*p* = 0.016).

No differences in the plasma levels of periostin (*p* > 0.05) and TSLP (*p* > 0.22) were observed among the asthma, COPD and ACO patient groups (Fig. 1c and d).

### Plasma NGAL can differentiate ACO from asthma, and plasma YKL-40 can differentiate ACO from COPD

ROC curve analysis was carried out to evaluate the sensitivity, specificity and accuracy of the plasma biomarkers for the differential diagnosis of ACO from asthma and COPD (Table 2). The area under the curve (AUC) showed that only plasma YKL-40 could distinguish patients with features of ACO from COPD patients (ROC AUC = 0.7138), and only NGAL was useful in distinguishing patients with features of ACO from asthma patients (ROC AUC =0.7517). Additionally, both YKL-40 and NGAL could distinguish patients with COPD from the controls and those with asthma (ROC AUC > 0.7). Although TSLP could discriminate the control from the patient groups, it was not available for the differentiation among the three patient groups.

**Table 2:** Receiver-operating characteristic (ROC) analysis for plasma biomarkers

	ACO vs asthma	ACO vs COPD	ACO vs control	COPD vs asthma	COPD vs control	asthma vs control
YKL-40						
AUC (95% CI)	0.5712[0.4932–0.6492]	0.7138[0.6496–0.7781]	0.8225[0.7523–0.8926]	0.7948[0.7369–0.8527]	0.8901[0.8428–0.9373]	0.8360[0.7639–0.9082]
Optimal threshold ng/ml	> 13.14	< 12.61	> 8.368	> 12.90	> 11.3	> 8.33
Sensitivity/specificity	31.37%/95.16%	73.47%/67.65%	80.39%/78%	71.43%/93.55%	80.27%/94.00%	88.71%/78%
NGAL						
AUC (95% CI)	0.7517 [0.6826–0.8207]	0.5936[0.5237–0.6636]	0.6859[0.5943–0.7775]	0.8000[0.7469–0.8530]	0.7537[0.6781–0.8294]	0.5063[0.3948–0.6178]
Optimal threshold ng/ml	< 104.7	> 179.1	< 87.23	> 104.3	> 89.90	> 75.43
Sensitivity/specificity	92.74%/58.82%	31.29%/93.14%	58.00%/76.47%	60.54%/92.74%	60.00%/79.59%	50.00%%/70.16
POSTN						
AUC (95% CI)	0.5471[0.4713–0.6229]	0.5224[0.4502–0.5945]	0.5062[0.4089–0.6035]	0.5691[0.5006–0.6376]	0.5343[0.4505–0.6181]	0.5440[0.4495–0.6385]
Optimal threshold ng/ml	> 89.87	< 112.1	> 66.29	< 90.45	> 66.29	> 85.92
Sensitivity/specificity	79.8%/33.3%	60.54%/52.94%	(92.00%/17.65%)	43.54%/79.84%	92.00%/23.81%	30.00%/82.26%
TSLP						
AUC (95% CI)	0.5474[0.4682–0.6266]	0.5462[0.4729–0.6195]	0.8733[0.8118–0.9348]	0.5230[0.4534–0.5926]	0.8824[0.8210–0.9438]	0.9191[0.8550–0.9833]
Optimal threshold ng/ml	> 5.044	> 4.012	> 3.665	> 3.568	> 3.522	> 3.568
Sensitivity/specificity	91.94%/26.47%	44.90%/71.57%	86.00%/83.33%	19.05%/100%	82.00%/83.67%	84.00%/100%

*Abbreviation*: *AUC* area under the curve, *YKL-40* chitinase-3-like protein 1, *NGAL* neutrophil gelatinase-associated lipocalin, *POSTN* Periostin, *TSLP* Thymic stromal lymphopoietin

### Relationships between the plasma biomarkers and subject characteristics

All the study subjects were pooled as a whole in order to analyze the possible correlations between the level of plasma biomarkers and subject characteristics including lung function and HRCT (Table 3). Age and smoking were found to correlate positively with plasma concentrations of YKL-40, NGAL and TSLP(*r* > 0.11, *p* < 0.02). The measures of lung function showed negative correlations with the levels of YKL-40, NGAL and TSLP(*r* < − 0.18, *p* < 0.0001), and the measures of the bronchodilator response showed negative correlations with the levels of YKL-40 and NGAL and positive correlations with the levels of TSLP(*r* < − 0.192, *p* < 0.0001). The percent of emphysema (% LAA-950) was found to be positively correlated with the levels of YKL-40 and NGAL (*r* > 0.377, *p* < 0.0001). Blood eosinophil counts were found positively correlated with TSLP, while blood neutrophil counts and serum creatinine positively correlated with NGAL. In our present study, no correlation was established between the plasma periostin level and measures of lung function (*r* = 0.086, *p* > 0.078), the bronchodilator response (*r* < 0.086, *p* > 0.075), however, a weak but statistically significant negative correlation with the percent of emphysema and blood neutrophil counts was found (*r* > − 0.101, *p* < 0.037).

**Table 3:** Correlations between plasma biomarkers and subject characteristics, lung function, bronchodilator response and the emphysema percent

	YKL-40	NGAL	PTON	TSLP
Age,years	0.275***	0.297***	−0.043	0.252***
BMI,kg/m^2^	−0.16**	−0.094	0.043	0.203***
smoking,park-years	0.388***	0.353 ***	−0.041	0.231***
SGRQ scores	0.390***	0.359***	−0.164**	−0.042
FVC % pred	−0.588***	−0.253***	0.043	−0.267***
FEV1% pred	−0.673***	− 0.348***	0.01	− 0.258***
FEV1/FVC,%	−0.595***	− 0.401***	0.081	− 0.174***
Change in FEV1, ml	−0.321***	− 0.241***	0.087	0.203***
%Change in FEV1, %	− 0.241***	−0.192***	0.077	0.252***
% LAA-950,%	0.482***	0.377***	−0.101*	0.076
Blood eosinophil, 10^8^/L	0.093	−0.09	0.04	0.114*
Blood neuttrophil, 10^9^/L	0.09	0.227***	−0.104*	0.081
Serum creatinine,umol/L	0.055	0.191 ***	− 0.011	0.091

Note: *: *p* < 0.05; **: *p* < 0.01; ***: *p* < 0.001

*Abbreviations*: *YKL-*40 chitinase-3-like protein 1, *NGAL* neutrophil gelatinase-associated lipocalin, *POSTN* Periostin, *TSLP* Thymic stromal lymphopoietin, *BMI* body mass index, *SGRQ* St George’s Respiratory Questionnaire, *FVC* forced vital capacity, *FEV1* forced expiratory volume in 1 s, *% pred* % predicted, *ΔFEV1* Change in FEV1, *SGRQ* St George’s Respiratory Questionnaire, *%LAA-950* The percentage of the low attenuation area below − 950 HU

### Multivariate stepwise analysis

Because the Spearman’s rank analysis above implied that plasma YKL-40, NGAL and TSLP levels are affected by several factors, we further performed multivariate stepwise analysis to ensure that the relationship between disease group and plasma biomarkers could be assessed after adjustment for potentially confounding variables (Table 4). Only variables with a *p* < 0.1 were retained in a stepwise analysis procedure.

**Table 4:** Multivariate stepwise regressions analysis of all subjects with each plasma biomarker as the dependent variable

YKL-40	β	T	*p*-value
FVC % pred,%	−0.314	−6.529	< 0.0001
FEV1%, pred	−0.301	−5.007	< 0.0001
% Change in FEV1, %	−1.497	−11.398	< 0.0001
FEV1/FVC,%	−0.325	−4.716	< 0.0001
Group, ACO vs. control	0.686	5.332	< 0.0001
Group, ACO vs. asthma	0.336	6.337	< 0.0001
Group, ACO vs. COPD	−1.014	−10.237	< 0.0001
NGAL			
% LAA-950,%	0.22	5.191	< 0.0001
% Change in FEV1, %	−0.172	− 2.688	< 0.0001
Group, ACO vs. asthma	0.24	4.251	< 0.0001
Group, ACO vs. COPD	−0.419	−7.686	< 0.0001
TSLP			
Group, ACO vs. control	0.374	8.262	< 0.0001

*Abbreviations*: *YKL-40* chitinase-3-like protein 1, *NGAL* neutrophil gelatinase-associated lipocalin, *TSLP* Thymic stromal lymphopoietin, *FVC* forced vital capacity, *FEV1* forced expiratory volume in 1 s, *% pred* % predicted, *ΔFEV1* Change in FEV1, *SGRQ* St George’s Respiratory Questionnaire, *%LAA-950* The percentage of the low attenuation area below − 950 HU

Because the Spearman’s rank analysis above implied that plasma YKL-40, NGAL and TSLP levels are affected by several factors, we further performed multivariate stepwise analysis to examine the effects of these independent variables age, BMI, smoke, lung function, bronchodilator response, the percent of emphysema (% LAA-950) and the studied disease categories (ACO vs. asthma, ACO vs. asthma and ACO vs. COPD) on the plasma levels of YKL-40, NGAL and TSLP (Table 4). PlasmaYKL-40 levels were significantly negatively associated with, in descending order of importance, change in FEV1 (%), ACO vs. COPD, FEV1/FVC %, FVC % predicted, and FEV1% predicted. Plasma NGAL levels were positively associated with the extent of emphysema, ACO vs. asthma, and negatively associated with change in FEV1 (%), ACO vs. COPD. Plasma TSLP levels were only found positively associated with ACO vs. control.

## Discussion

Our present study explored plasma levels of 4 biomarkers associated with asthma, COPD and their possible correlation with lung function, bronchodilator response and radiographic imaging in asthma and COPD patients and patients with features of ACO. Our results demonstrate that patients with features of ACO displayed distinct features, they had lower plasma YKL-40 than COPD patients and a moderate elevated plasma level of NGAL compared with asthma patients; had an intermediate degree of airflow obstruction, bronchodilator response and emphysema between patients with COPD and asthma. Furthermore, negative correlation between plasma YKL-40 and lung function and the bronchodilator response, and positive correlation between plasma NGAL and the extent of emphysema were observed. Therefore, ACO was an intermediate condition between COPD and asthma with distinct pathophysiological features. Plasma YKL-40 and NGAL are promising candidates for distinguishing ACO from asthma and COPD.

One of the main findings of our present study is that the plasma YKL-40 levels were significantly lower in asthma patients and patients with features of ACO than in COPD patients. YKL-40 was originally shown to be a key regulator in Th2-mediated inflammation and tissue remodeling [26, 27]. Clearly increased YKL-40 levels were found in the plasma and lungs of asthma patients, where they correlated with disease severity and measures of airway remodeling [27–29]. Our present study showed a more elevated level in COPD patients than in patients with asthma and patients with features of ACO. This result seemed to conflict with the opinion that YKL-40 is a Th2-type biomarker. In fact, more pronounced upregulation of YKL-40 in COPD patients than in asthma patients was reported in Jame’s recent studies [24]. This is consistent with our present study and it suggest that YKL-40 is associated with COPD-related pathophysiology in addition to Th2-type inflammation; thus, it may be a potential biomarker to clinically identify ACO [30] and more investigations are needed in the future to elaborate the role of YKL-40 in the pathogenesis of chronic airway diseases.

Previous studies have indicated that plasma YKL-40 levels are positively correlated with subepithelial basement membrane thickness and extracellular matrix deposition in the airway walls, suggesting its important role in airway remodeling [31, 32]. Airway remodeling is a major cause of airway narrowing and airflow limitation, as well as the impaired response to bronchodilators [33]. Our results demonstrated that the plasma YKL-40 levels in patients with features of ACO and asthma patients were lower than those in COPD patients and simultaneously higher than those in control subjects. Furthermore, there was a clear negative correlation between the plasma YKL-40 levels and lung function as well as the bronchodilator responsiveness, suggesting that moderately elevated levels of plasma YKL-40 might be a characteristic feature of ACO.

The differences in plasma YKL-40 levels between patients with features of ACO and those with asthma were not statistically significant in our present study, although the ACO groups indeed had a higher median value. One possible reason may be that most asthma subjects were severe and therapy-resistant in-patients enrolled from exclusively teaching hospitals or district general hospitals in our study, and it was widely demonstrated that the plasma YKL-40 levels in those patients were notably higher than those in the mild to moderate, well-controlled asthma patients [29].

NGAL, a member of the lipocalin family released from neutrophils [34] upon activation. As a widely accepted marker of neutrophilic inflammation, significantly higher plasma levels of NGAL were observed in patients with features of ACO than in asthma patients, and subsequent ROC curve analysis confirmed its role in differentiating ACO from asthma. ACO is common in asthma patients with long-term smoking and recurrent infection, particularly in older populations [33]. As a well-established neutrophil-derived inflammatory molecule, NGAL is highly associated with inflammation and emphysema in smoking- and chronic infection-related diseases [21].

Our experimental findings showed that the plasma NGAL levels were lower in the overlapped patients than in patients with COPD, highlighting that plasma level of NGAL may also exert an important function in differentiating ACO from COPD. Additionally, in terms of the pathophysiological mechanism, in contrast to the dominant neutrophilic airway inflammation in COPD, inflammation in ACO seems to be more complex and may be predominantly driven by both neutrophils and eosinophils [34].

Increasing studies have demonstrated that emphysema is a characteristic imaging manifestation of patients with features of ACO. Compared with COPD patients, patients with features of ACO usually have a lower degree of emphysema [35, 36]. Our study also found less emphysema in patients with features of ACO than in COPD patients, suggesting that the degree of emphysema can help characterize ACO as a distinct clinical entity from COPD. Interestingly, our data demonstrated that among the 4 biomarkers chosen in our study, only the plasma NGAL level showed a significantly positive correlation with the percent of emphysema.

Indeed, the increased NGAL level has been proven to be a valid and reliable molecular marker that correlates with the extent of emphysema associated with smoking [34]. Therefore, plasma NGAL and other neutrophil-related biomarkers may somewhat reflect the underlying pathophysiological changes of ACO.

TSLP has been identified as a “master switch” for allergen-induced inflammation through the activation of dendritic cells to induce type 2 inflammation [37, 38]. However, our present study demonstrated similar increased plasma TSLP levels in patients with asthma, COPD, and patients with features of ACO with those in control subjects. It was very recently reported that TSLP measurements were highly dependent on the ELISA kits used; TSLP in induced sputum is a more reliable biomarker than plasma TSLP [39]. However, a significant correlation between plasma TSLP and airway obstruction, as well as airflow reversibility, was indeed displayed in our results. Thus, further investigation with other biological samples, like exhaled breath condensate, induced sputum, and bronchoalveolar lavage fluid using different ELISA assays, is needed to explore the potential role of TSLP in identifying ACO.

Periostin is an extracellular matrix protein expressed in mesenchymal cells and bronchial epithelial cells [40]. As a downstream molecule of IL-4 and IL-13, it has been proposed as an important biomarker of the Th2-high eosinophilic phenotype of asthma [41]. No significant differences were found in the plasma periostin levels among subjects with control, asthma, COPD and subjects with features of ACO or in the correlation between the plasma periostin levels with lung function, airflow reversibility, or percent of emphysema in our study, suggesting that plasma periostin might not be a sensitive Th2-type biomarker for ACO screening.

## Conclusions

Plasma YKL-40 is a promising candidate for distinguishing ACO from COPD, while plasma NGAL may be a valuable biomarker for distinguishing ACO from asthma. These were beneficial for the early recognition of ACO and subsequent targeted intervention, which is important given the rapid progress and worse prognosis of this disease. Further studies are required to investigate the possible role of these biomarkers in the pathogenesis of ACO. Regarding the three conventional Th2-type biomarkers chosen in our study, only YKL-40, which is now recognized as a non T-helper cell type 2 biomarker, was found to play a role in screening out ACO; other Th2-type biomarkers need to be explored in the future.

## Abbreviations

ACO: Asthma–Chronic Obstructive Pulmonary Disease Overlap
AUC: Area Under the Curve
BMI: Body mass index
CI: Confidence interval
COPD: Chronic obstructive pulmonary disease
CT: Computed tomography
FEV1: Forced expiratory volume in 1 s
FEV1/FVC: The ratio of the forced expiratory volume in the first 1 s to the forced vital capacity of the lungs
FVC: Forced vital capacity
GINA: Global Initiative for Asthma
GOLD: Global Initiative for Chronic Obstructive Lung Disease
HRCT: High-resolution computed tomography
NGAL: Neutrophil gelatinase-associated lipocalin
ROC: Receiver operating characteristic
SD: Standard deviation
SEM: Standard Error of Mean
SGRQ: St. George’s Respiratory Questionnaire
Th2: T-helper 2
TSLP: Thymic stromal lymphopoietin
YKL-40: Chitinase-3-like protein 1
